# Study on edge villages based on cross-region healthcare-seeking behavior

**DOI:** 10.1186/s12942-025-00426-6

**Published:** 2025-12-11

**Authors:** Lulu Liu, Yu Xiao, Haiqing Xiang, Jia Liang

**Affiliations:** 1 Academy of Cultural Heritage and Creativity of Jinan University, Guangzhou, 510000 China; 2 The First Affiliated Hospital of Guangdong Pharmaceutical University, Guangzhou, 510000 China; 3 School of Mechanics and Constructions Engineering, Jinan University, Guangzhou, 510000 China

**Keywords:** Cross-region healthcare-seeking behavior, Cross-region healthcare, Edge village, Remote and underdeveloped areas, Primary rural healthcare

## Abstract

**Background:**

This study addresses spatial disparities in rural healthcare by introducing the concept of edge village —communities where a misalignment between administrative boundaries and functional hospital service areas (HSAs) leads to prevalent cross-region healthcare-seeking. This concept, grounded in edge-effect theory, provides a novel perspective for analyzing healthcare resource mismatches.

**Methods:**

Using Liannan Yao Autonomous County, Guangdong Province, as a case study, we employed complex network community detection to delineate HSAs and identify edge villages. An institution-behavior-space integrative framework was applied, combining literature analysis and field surveys to establish a multidimensional factor system. Key indicators were selected via Elastic Net regression, and their impact mechanisms were analyzed using mixed logit models.

**Results:**

Edge villages were systematically identified, revealing significant misalignment between actual healthcare service areas and administrative divisions. Key factors driving cross-region healthcare-seeking included service accessibility, resource quality, and patient mobility patterns. The proposed framework effectively interprets spatial disparities through the lens of edge villages.

**Conclusions:**

The edge village concept offers a new micro-analytic unit and a transferable framework for understanding rural healthcare misallocation. It provides policymakers with an evidence-based tool to pinpoint underserved areas and formulate tailored governance strategies, thereby improving resource allocation fairness and efficiency and fostering inclusive regional development.

## Introduction

Public services—particularly healthcare resources—are unevenly distributed—a long-standing global challenge that disproportionately burdens rural and remote communities across developed and developing contexts. Owing to persistent shortages in local provision, residents in these areas are often compelled to seek care beyond their administrative area—a phenomenon often termed cross-region healthcare [18, 73]. In many settings, resources are concentrated in urban centers, and well-intended accessibility policies can inadvertently exacerbate spatial inequalities. This pattern not only reflects individual service-seeking behavior but also reveals the complex interdependencies among resource allocation, rigid administrative boundaries, and supply–demand imbalances [76]. Accordingly, a focused examination of cross-region healthcare in remote and underdeveloped areas—and of the functional care regions that emerge from it—is crucial to reassessing and improving rural healthcare coverage and efficiency.

Cross-region healthcare has been examined from various perspectives. Some studies focus on patients bypassing primary care in favor of higher-tier services (Hanlon and Skedgel 2006 ), while others examine mobility across administrative or economic boundaries [59]. Studies at the national level have examined cross-region healthcare behaviors and their influencing factors [68, 74]. For example, [17]. identified four dimensions of patient mobility: availability, familiarity, affordability, and perceived quality. In Europe, cross-border healthcare has attracted attention due to policy reforms that facilitate mobility across sovereign states [7, 18, 40]. While cross-border healthcare fundamentally differs from cross-region healthcare—which addresses patient mobility across administrative divisions within a single country—European studies nonetheless offer methodological insights. In the Chinese governance context, however, policy attention centers on cross-region healthcare, shaped by hierarchical medical systems, uneven resource distribution, and interprovincial health insurance policies. Despite these developments, empirical research on cross-region healthcare remains limited. A key challenge is data access, particularly in less developed and remote areas [43, 53]. Moreover, most studies focus on large-scale regional patterns, while research on smaller-scale contexts and primary healthcare systems remains limited [8, 71].

Cross-region healthcare is closely linked to the delineation of hospital service areas (HSAs), which are influenced by patient mobility and disparities in healthcare resource [5, 15]. Methods for delineating HSAs can be categorized into three main approaches: (1) spatial analyses based on observed healthcare data (e.g., patient flows), including the Dartmouth method, the Huff model, and network community detection (e.g., Louvain/Leiden), but these approaches often face data-access constraints and operate at relatively coarse spatial scales [20, 29, 66],(2 spatial-interaction models grounded in potential accessibility—most notably the gravity model and its special case, the two-step floating catchment area (2SFCA method—are also widely used [41, 64, 72], In recent years, researchers have incorporated nonspatial factors into the 2SFCA framework, thereby enhancing the explanatory power of accessibility assessments [54]. Nevertheless, fully integrating socioeconomic attributes, behavioral preferences, and other multidimensional nonspatial components within a unified framework remains an open question,and (3) fixed-radius delineation methods, such as the "living circle" approach, have been criticized for their rigidity and limited applicability [55, 65]. Existing research predominantly focuses on urban and large-scale settings, whereas rural and small-scale contexts remain underrepresented. Future research should develop more fine-grained and adaptable HSA delineation methods, particularly suited to data-sparse rural contexts.

In China, HSAs are primarily delineated based on administrative boundaries. However, in high-demand areas where resources are scarce, these boundaries—while facilitating resource organization—can also lead to fragmentation [58, 75]. Townships serve as both governance units and primary providers of primary care, potentially limiting residents’ choices and hindering equitable resource allocation [36]. Consequently, cross-region healthcare has become an adaptive strategy for rural residents. In recent years, policy reforms and the relaxation of health-insurance rules have made care-seeking outside one’s administrative jurisdiction more convenient, thereby intensifying cross-region healthcare. In this study, we define cross-region healthcare as residents seeking medical services beyond their home administrative jurisdiction. At he county level, this specifically refers to travelling across township or county borders to access healthcare facilities in other regions. This study primarily focuses on the phenomenon where patients, due to issues such as the imbalance of medical resources and the unreasonable delineation of existing HSAs, seek medical treatment in regions with better healthcare conditions or more convenient transportation, while still within the same insurance coverage area as neighboring regions.

Within the broader phenomenon of cross-region healthcare, villages whose residents frequently seek care beyond local administrative borders warrant particular analytical attention. We define such settlements as “edge villages”, namely communities where misalignment between administrative boundaries and functional HSAs induces widespread cross-region care-seeking. The concept is grounded in the core tenets of edge-effect theory. Originally developed in ecology, the edge effect denotes distinctive characteristics and behavioral patterns that arise in boundary zones where interacting systems meet [13, 44]. The notion has since been applied in sociology and medical geography to characterize resource configurations and salient phenomena in peripheral areas [6, 51]. In the healthcare domain, the edge effect manifests as distinctive care experiences and access conditions encountered when residents cross administrative borders to obtain services [16]. Prior studies indicate that overlooking edge effects may lead to biased inferences about the spatial distribution of healthcare resources and access to services [26, 61].

Within this theoretical lens, the proposed concept of “edge villages” captures the spatial pattern of care-seeking that arises when HSAs are misaligned with administrative jurisdictions. Situated along administrative borders, these villages are often loci of intensified cross-region healthcare, offering a critical entry point for examining tensions between the spatial organization of medical resources and institutional boundaries. Typically located at the periphery of the healthcare system—and often at the margins of economic, political, and social life—such settlements provide a revealing vantage point on spatial inequalities in healthcare resources.

To systematically explicate the concept of edge villages, this study adopts an overarching perspective grounded in edge-effect and boundary theories, and integrates behavioral and spatial dimensions into a coherent analytical framework. First, the boundary lens provides the foundation for analyzing the institutional origins of edge villages, emphasizing systemic segmentation and resource-allocation distortions induced by administrative borders and establishing the premise for tracing their roots through the institution–space contradiction. Second, on the behavioral dimension, we draw on Andersen’s Behavioral Model of Health Services Use. Since its introduction in 1968 and subsequent revisions, this model has become a mainstream framework for analyzing health-service utilization; on the demand side, it highlights the joint influence of predisposing characteristics, enabling resources, and perceived need on care choices, providing a solid micro-level basis for understanding cross-region care decisions [49, 62]. Finally, on the spatial dimension, we employ accessibility theory rooted in geography and further elaborated by Penchansky and Thomas [48]. Moving beyond simple geographic distance, this perspective focuses—on the supply side—on the spatial distribution and allocative efficiency of healthcare resources, and encompasses multidimensional “fit” (e.g., availability, affordability, acceptability). This conceptualization is complemented by Jean-Frederic Levesque’s patient-centered perspective on healthcare accessibility [30], which emphasizes the interface between supply-side features and individuals’ abilities to perceive, seek, reach, pay, and engage. Together, these perspectives provide a combined spatial-social basis for assessing supply–demand matching. By integrating institutional perspectives, behavioral choice, and spatial constraints, the framework seeks to explain—at the village scale—the mechanisms through which edge villages form and the dynamic processes of resource mismatch that underlie their emergence.

Guided by the foregoing theoretical framework, this study undertakes an identify-explain-apply sequence. First, we apply complex network algorithms—specifically the Louvain community detection algorithm—to county-level patient-flow data to identify “edge villages,” defined as locations where functional HSAs are misaligned with administrative jurisdictions, thus operationalizing the concept as a measurable empirical construct. Second, we construct a multidimensional indicator system spanning demand-, supply-, and relational/connectivity dimensions and employ Elastic Net together with a mixed logit model to identify key drivers and pathways of influence. Finally, drawing on the empirical results, we propose evidence-informed governance strategies tailored to edge villages.

Through this research design, the study aims to advance both theory and practice. Theoretically, by introducing edge villages as a micro-analytic unit and integrating a multidisciplinary perspective, we advance a framework for understanding the spatial misallocation of rural healthcare resources. Practically, combining precise identification with mechanism analysis, we provide empirical evidence to inform the optimization of county-level healthcare resource allocation and to promote cross-region collaborative governance. Taken together, the study offers a comprehensive analytical pathway for diagnosing the edge-village problem and a transferable paradigm for related research in comparable settings.

## Materials and methods

**Fig. 1:**
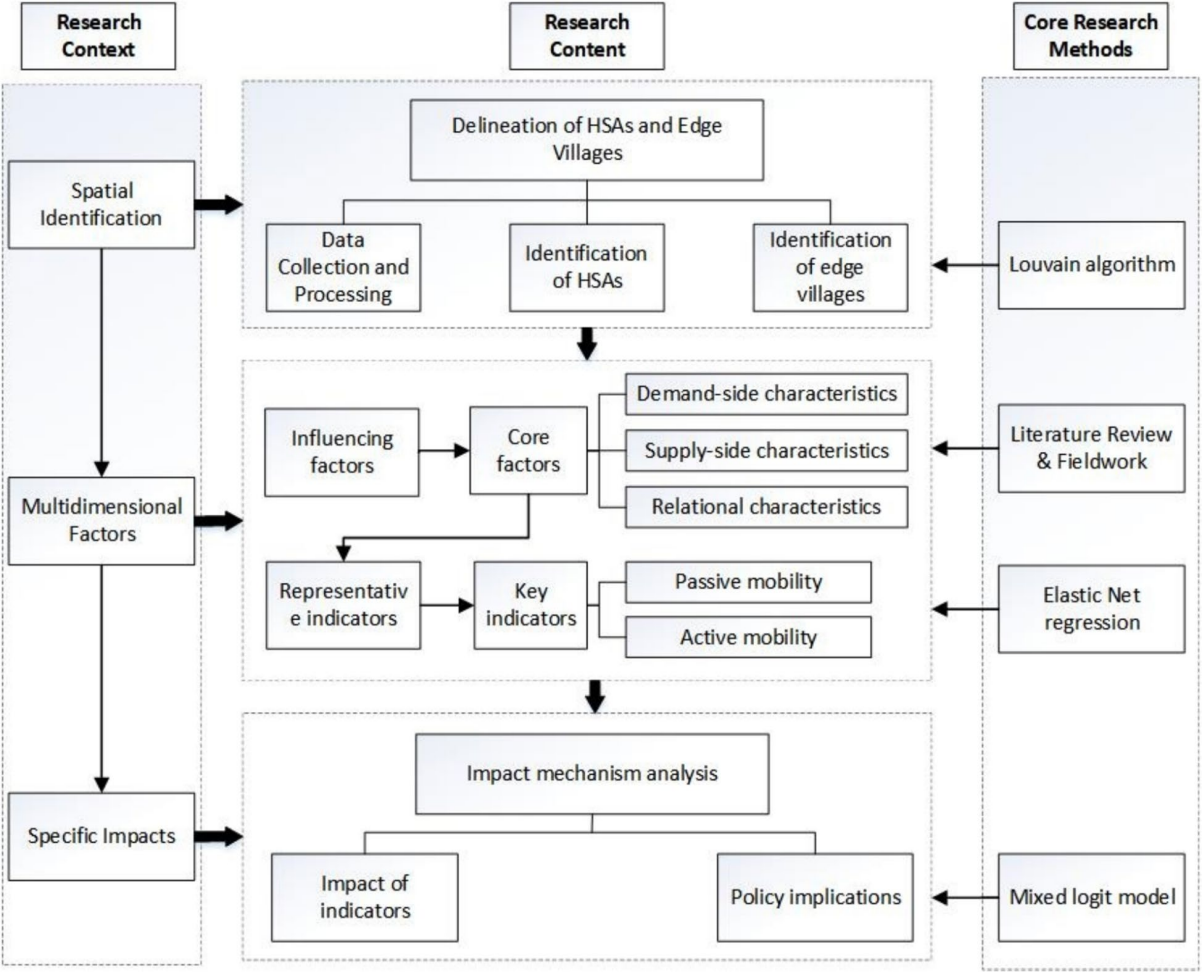
Research design and analytical framework

Figure 1 presents an overview of the methodological workflow linking data collection and processing, Louvain-based delineation of HSAs and edge villages, Elastic Net variable selection, and mixed logit mechanism analysis, culminating in policy implications.

### Study area

**Fig. 2:**
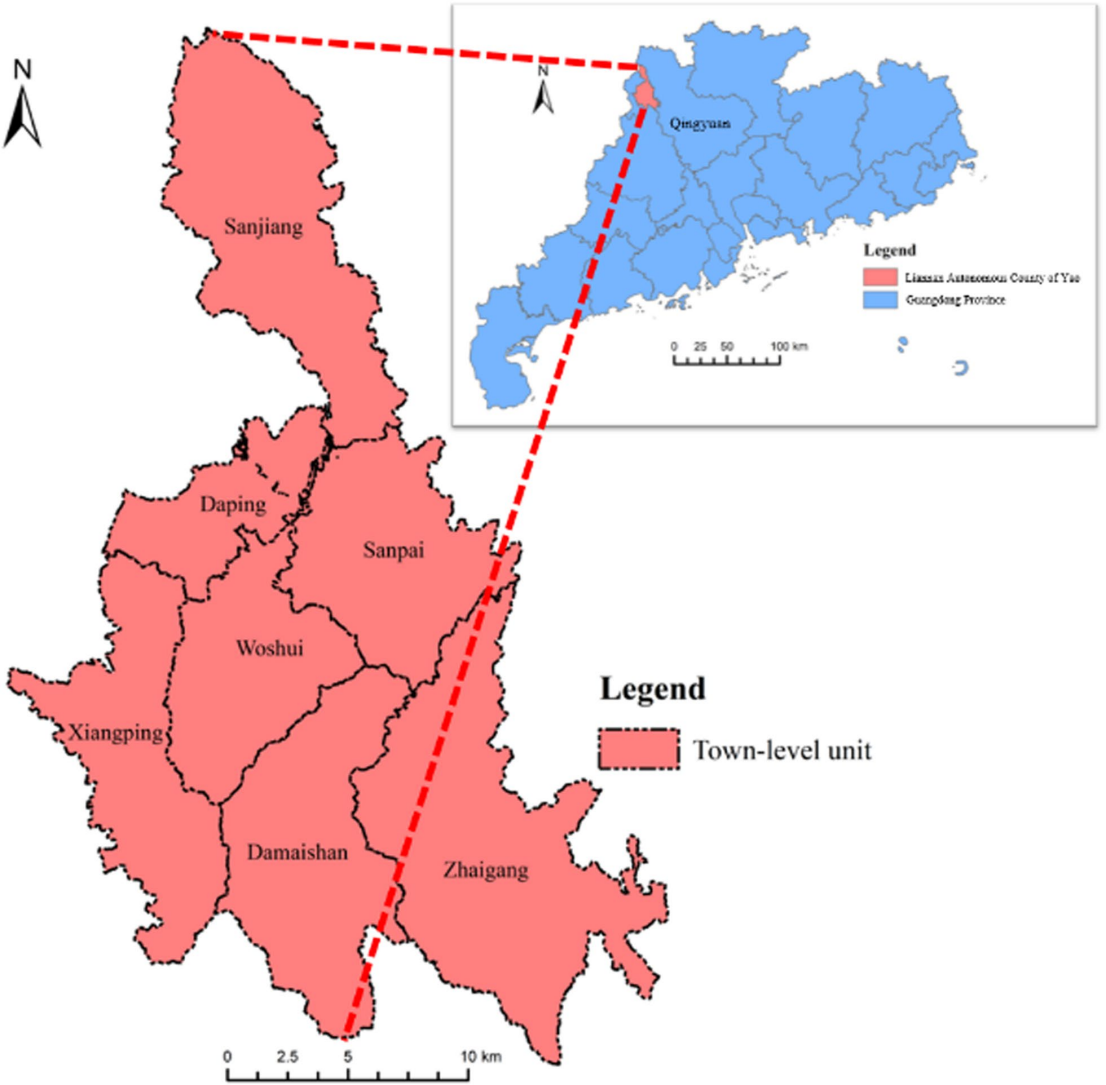
Location map of the study area

Liannan Yao Autonomous County (Liannan County), located in the mountainous region of Qingyuan City, Guangdong Province, is a key transportation hub at the junction of three provincial boundaries (Fig. 2). It borders Lianzhou City to the northeast, Yangshan County to the southeast, Huaiji County to the south, Lianshan Zhuang and Yao Autonomous County to the west, and Jianghua Yao Autonomous County in Hunan Province to the northwest. Covering 1,306 km2, it has 7 towns, 71 village-level committees, and a population of 180,000, including 135,000 permanent residents. Ethnic minorities, primarily the Yao, constitutes 58% of the population. Liannan is the largest autonomous county in Guangdong by minority population, preserving rich Yao traditions and earning the name "Hundred-Mile Yao Mountain" for its historical and cultural significance.

**Fig. 3:**
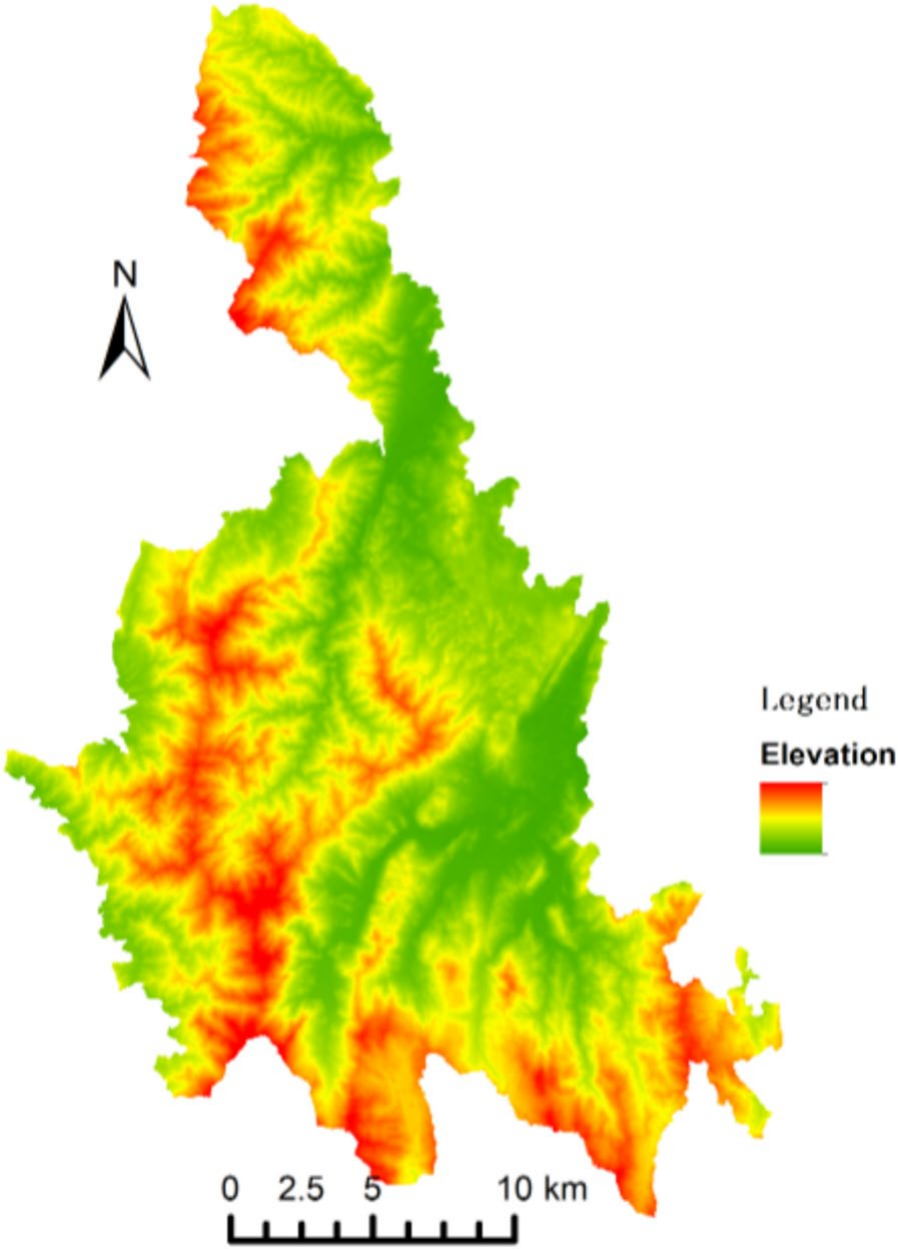
Digital elevation model

**Fig. 4:**
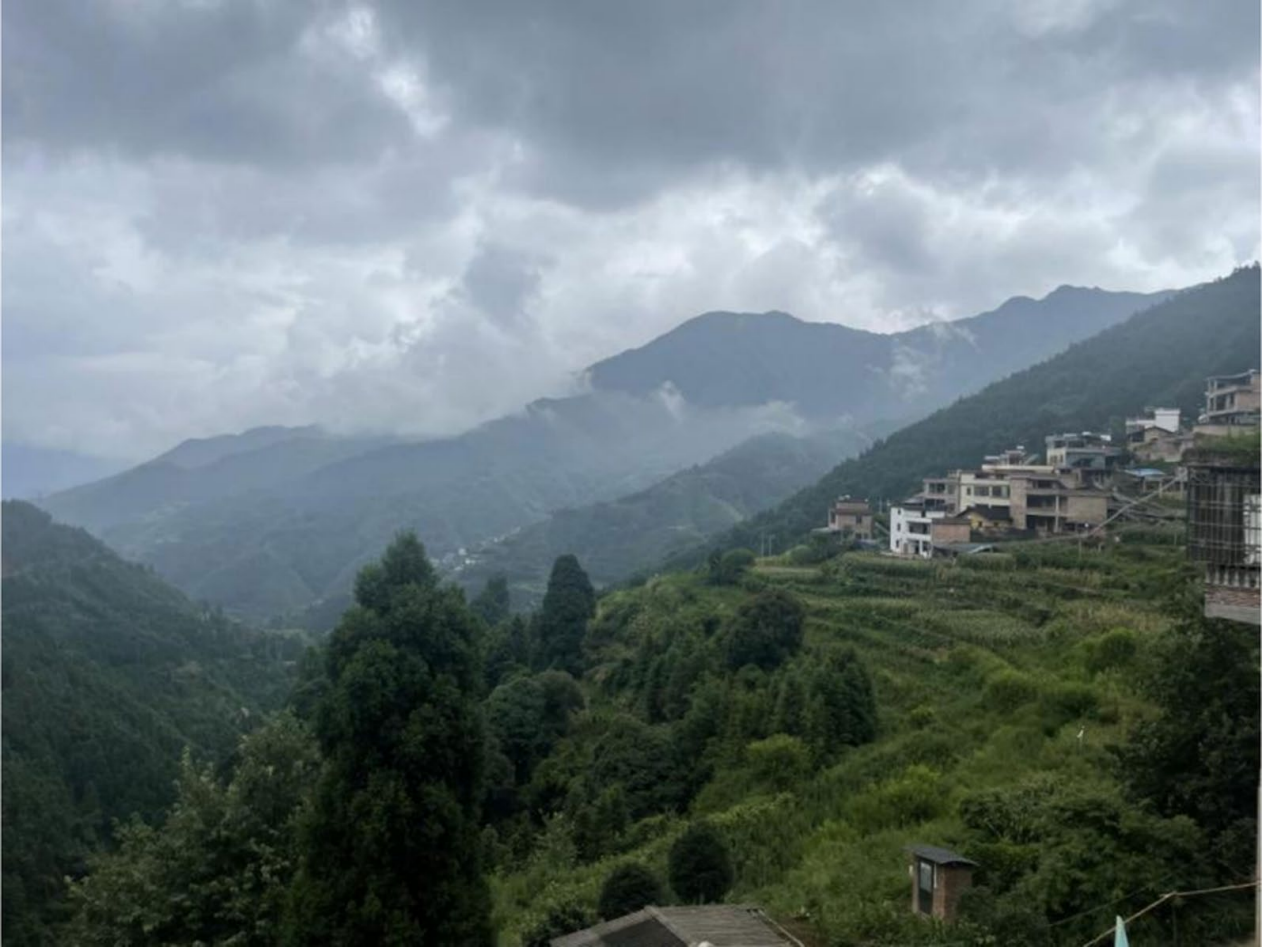
Typical topography of liannan county, with many villages built on hillsides or mountaintops

Liannan County has a narrow, elongated terrain, spanning 45 km at its widest in the northwest and 71 km north–south. Its rugged landscape includes widely dispersed hills and 161 peaks over 1,000 m. Figure 3 shows a digital elevation model (DEM), highlighting geographic challenges for governance. Poor transportation and mountainous terrain once made Liannan one of Guangdong’s poorest regions (Fig. 4). However, its proximity to prosperous Yao-inhabited towns in Lianshan County, along with cultural ties, attracts residents for healthcare, fostering cross-county patient flow.

### Analytical methods

#### Community detection in complex networks

This study applies network community detection to analyze HSAs in Liannan County. The healthcare-seeking network is modeled as a graph, where nodes represent patient locations, edges depict healthcare-seeking behavior, and weights indicate patient flow intensity or outflow ratio. This network illustrates the demand–supply interplay in healthcare [4, 24].

Community detection algorithms vary based on optimization objectives. Newman and Girvan introduced modularity, which maximizes intra-community connections while minimizing inter-community edges [45]. This study adopts the Louvain algorithm for its efficiency and hierarchical stability, making it ideal for analyzing healthcare-seeking networks in densely populated or large regions. The Louvain algorithm has two main stages

##### Local node movement stage

Each node starts in its own community. The algorithm iterates through neighbors, calculating modularity gain for potential community shifts, selecting the highest gain.

##### Network aggregation stage

Communities are collapsed into nodes, forming a new network. Edge weights update, and the process repeats until modularity no longer improves.

This iterative process yields optimal community partitioning. The algorithm was implemented using Python’s igraph package.

### Elastic net regression

Elastic Net regression is a regularized linear modeling approach that combines L1 and L2 penalties, making it well suited to datasets with many irrelevant or highly correlated predictors [78]. This approach combines the sparsity of the least absolute shrinkage and selection operator (LASSO) with the stability of ridge regression, yielding variable selection via coefficient shrinkage. When multiple variables exhibit collinearity, the parameter λ adjusts balance between L1 and L2 regularization, improving the model’s performance compared to LASSO and Ridge regression. The principle behind this model is to minimize the following loss function to achieve optimal data fitting:

1 $$\begin{aligned}Minimize \frac{1}{2n}{\sum }_{i=1}^{n}\begin{array}{c}{\left({y}_{i}-{\beta }_{0}-{\sum }_{j=1}^{p}{\beta }_{j}{x}_{ij}\right)}^{2}\\+\lambda \left(\alpha {\sum }_{j=1}^{p}\left|{\beta }_{j}\right|+\frac{1-a}{2}{\sum }_{j=1}^{p}{{\beta }_{j}}^{2}\right)\end{array}\end{aligned}$$

where $n$ is the number of samples, $p$ is the number of features, ${y}_{i}$ is the observed value of the $i$-th sample, ${x}_{ij}$ is the value of the $j$-th feature for the $i$-th sample, ${\beta }_{j}$ is the coefficient of the $j$-th feature, $\lambda$ is the regularization parameter that controls the strength of the regularization, and $\alpha$ is the parameter that controls the relative contribution of the L1 and L2 regularization terms.

Elastic Net regression can shrink or zero-out irrelevant predictors, mitigate multicollinearity, and enhance model stability. Accordingly, we employ Elastic Net as the primary method for feature selection.

### Mixed logit model

Beyond identifying the formation of edge villages, we estimate a mixed logit model to quantify the utility effects of key determinants on community healthcare choices [22].

The mixed logit model is grounded in utility maximization theory and assumes that the utility of community $i$ residents choosing alternative $j$ is given by:

2 $$\begin{array}{c}{\text{U}}_{{\text{ij}}_{0}}={\text{V}}_{{\text{ij}}_{0}}+{\upvarepsilon }_{{\text{ij}}_{0}}\end{array}$$

where ${V}_{i{j}_{0}}$ is the observable utility and ${\varepsilon }_{i{j}_{0}}$ represents the unobservable factors. The linear form of the observable utility is expressed as:

3 $$\begin{array}{c}{V}_{i{j}_{0}}=ASC+{\beta }_{j}{X}_{ij}\end{array}$$

Here, $ASC$ is the alternative-specific constant, ${X}_{ij}$ represents the attributes of the healthcare choice, and ${\beta }_{j}$ denotes the corresponding parameters.

In this study, the selected variables are incorporated into the mixed logit model for parameter estimation to analyze the probability of patients choosing cross-region healthcare. The probability expression of the mixed logit model is:

4 $${P}_{i}\left(j\right)=\frac{\text{exp}\left({{x}{\prime}}_{i}{\beta }_{j}+{{z}{\prime}}_{ij}\upbeta \right)}{{\sum }_{m=1}^{J}\text{exp}\left({{x}{\prime}}_{i}{\beta }_{m}+{{z}{\prime}}_{ij}\upbeta \right)}$$

where ${P}_{i}\left(j\right)$ is the probability that community $i$ selects healthcare choice $j$, with ${{x}{\prime}}_{i}$ representing the community’s inherent attributes (e.g., demographic structure, location) that do not change across choices, and ${{z}{\prime}}_{ij}$ representing the attributes that vary by healthcare choice (e.g., travel distance, available resources).

The mixed logit model can simultaneously account for individual- and choice-specific characteristics, capture unobserved utility associated with the alternatives, and effectively handle heterogeneity in choice behavior. Therefore, this model is particularly suitable for analyzing complex community healthcare choices.

## Data

### Primary data

The data used in this study for the Louvain algorithm analysis were collected from the Early Cancer Screening Program of the Guangdong County-level Medical Community Tumor Prevention and Treatment Center, which was conducted at the Liannan People’s Hospital from April to May 2023. This project indirectly collected healthcare-seeking behavior information from residents participating in the early cancer screening through a questionnaire survey, resulting in 4,314 valid records of healthcare behavior flow data. The residents who participated in the survey were required to be at least 18 years old, have lived in Liannan County for over one year, and voluntarily participated in the screening and questionnaire filling. Given the relatively low frequency of healthcare utilization in rural areas, the collected data include not only actual healthcare-seeking behaviors but also self-reported healthcare intentions.

During the data processing stage, respondents were assigned to their corresponding administrative villages based on their reported residential locations. Data were then mapped to the townships or streets where the medical institutions are located according to actual or intended healthcare flow, transforming the data into OD (Origin–Destination) flow data (see Table 1). To enhance the stability and reliability of the analysis, healthcare flow data from administrative villages with fewer than 10 records were excluded to minimize sample bias and reduce uncertainty in the algorithm results. Additionally, some communities were merged into neighboring administrative villages to ensure consistency in calculations and enhance analysis quality.

**Table 1:** Example of patients’ actual or intended healthcare OD flow data

Respondent ID	Residential location	Healthcare (or intended healthcare) location
A1	Baimang village	Damaishan health center
A2	Baimang village	Damaishan health center
B1	Shimang village	Zhaigang central health center, Zhainan branch

The processed OD flow data (with personal information removed) were represented as flows from administrative villages to townships and were normalized to compute the proportion of flow from each administrative village to its respective township. These proportions were used as weight values in the community detection algorithm:

5 $$\begin{array}{c}{W}_{ij}=\frac{{D}_{ij}}{\sum_{j}{D}_{ij}}\end{array}$$

The final nodes, edges, and weights were stored in the NCOL file format and used as the input data for the Louvain algorithm to derive the spatial network structure of residents’ healthcare-seeking behaviors within the local healthcare service system.

### Auxiliary data

In addition to the primary data mentioned above, this study also utilizes supplementary datasets for visualizing results, including planning land use data provided by the Liannan Yao Autonomous County Natural Resources Bureau and a 2023 road network map of primary and secondary roads in Liannan County, which was extracted from AutoNavi Map. These datasets were primarily used for result visualization in ArcGIS (developed by Esri).

### Ethical approval and data authorization

This study was reviewed and approved by the Ethics Committee of the First Affiliated Hospital of Guangdong Pharmaceutical University [Ethics approval number: (2025) IIT (7)]. In addition, data collection was conducted under formal authorization from Liannan People’s Hospital and in compliance with county-level health-authority requirements. All patient-flow and geographic information used in this study were anonymized prior to analysis, and no identifiable personal data were included. Due to the retrospective nature of the study, which involved only secondary analysis of anonymized data, the Ethics Committee granted a waiver of informed consent. The research was conducted in accordance with the Declaration of Helsinki and its subsequent amendments.

## Results

### Delineation of HSAs and edge villages

#### Identification of HSAs

**Fig. 5:**
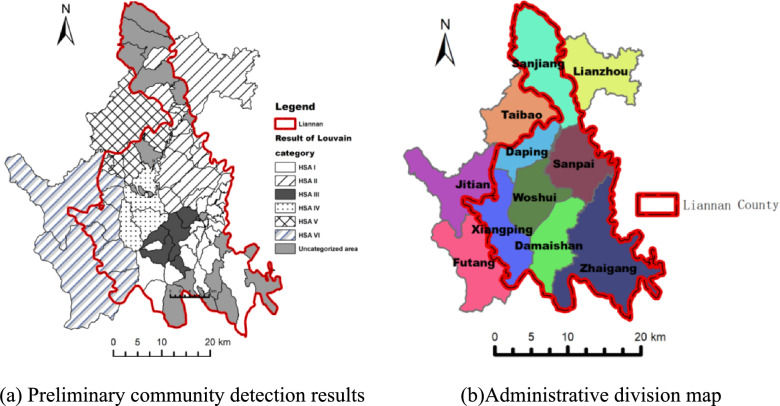
Comparison of preliminary community detection results and administrative divisions

This study applies the Louvain algorithm to compute the HSAs of Liannan County, where the initially detected communities serve as the preliminary form of HSAs. The Louvain algorithm was executed in two iterations: the first iteration identified 9 HSAs with a modularity of 0.4262051, and the second iteration identified 6 HSAs with a modularity of 0.4494736. According to Newman’s research, modularity values for most real-world networks typically range from 0.3 to 0.7, suggesting that the computed results fall within the expected range [45]. The HSAs detection results were imported into ArcGIS for visualization, as illustrated in Fig. 5a.

Although the Louvain algorithm does not explicitly optimize spatial contiguity, the initial HSA delineation was largely contiguous. notably, non-residential areas and residential areas with data missingness were left unassigned, resulting in isolated fragments. This is a common issue when using empirical patient-flow data for HSA delineation [61]. To improve assignment accuracy in the healthcare-seeking network, the following pre-specified, rule-based post-processing adjustments were implemented to address areas with insufficient data

##### Non**-**residentialareas (e.g. forest tracts)

Due to the absence of resident populations—and hence no healthcare flows—these areas were merged into adjacent administrative villages based on road-network contiguity and administrative jurisdiction.

##### Residential areas with extreme data sparsity (< 10 patient-flow records)

To mitigate uncertainty from small counts, supplementary field interviews were conducted with local health-station staff and primary-care physicians. Based on their knowledge of predominant care-seeking patterns, these villages were assigned to the HSA receiving the majority of observed flows.

**Fig. 6:**
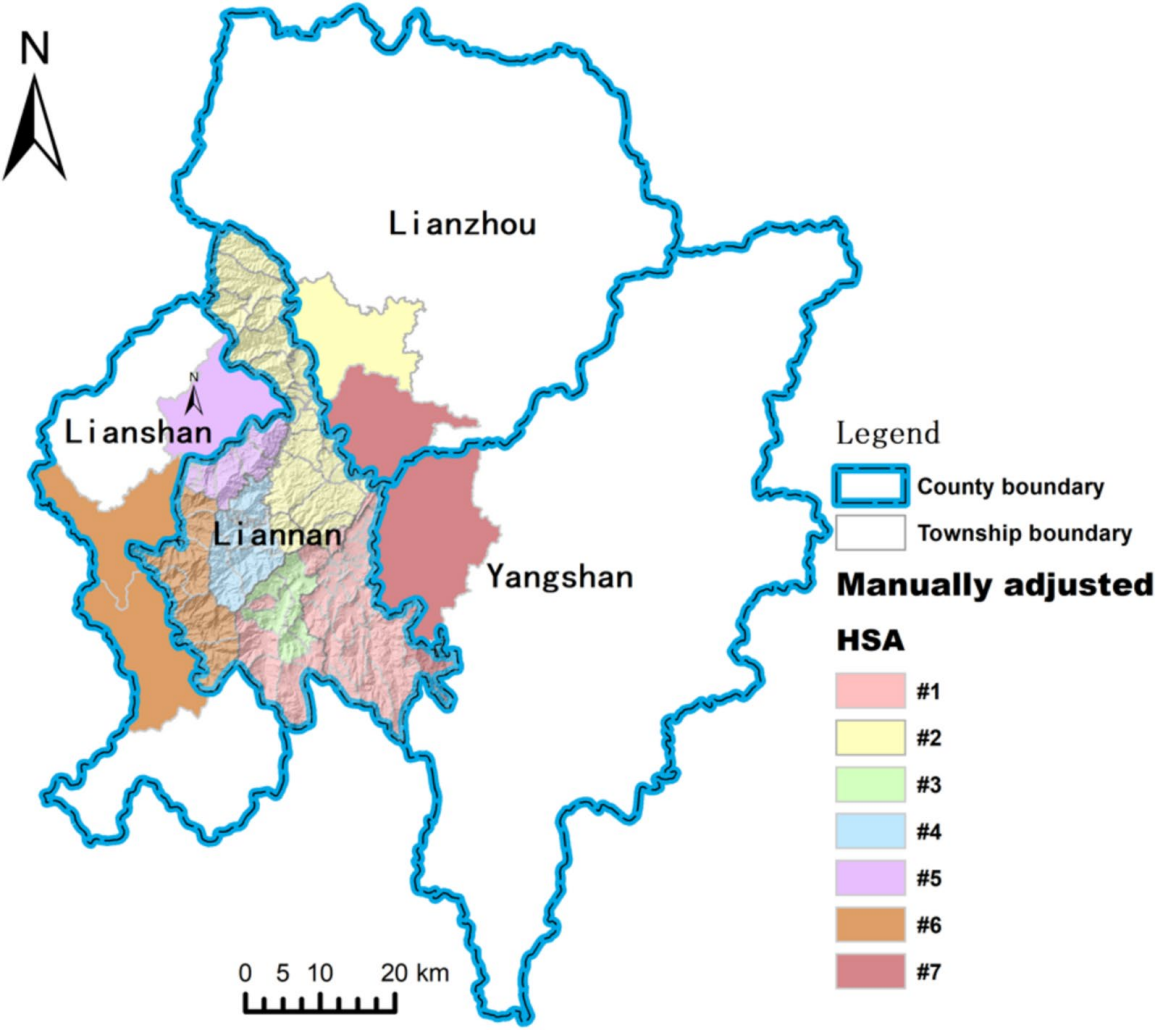
Community detection results after manual adjustments

After these modifications, all regions were successfully assigned to an HSA, and the results were visualized in ArcGIS, as illustrated in Fig. 6. Their structure exhibits the following characteristics:

#### Constraints and breakthroughs of administrative boundaries

Currently, the designated HSAs for primary healthcare institutions are delineated based on administrative boundaries. However, the HSAs derived from actual healthcare-seeking behaviors of residents do not fully align with the initially designated HSAs. The computed HSAs generally conform to administrative boundaries but extend beyond them in certain areas. This phenomenon is particularly evident along the borders of Xiangping Town, where residents exhibit diverse healthcare-seeking patterns.

#### Super-HSA phenomenon

The community detection results reveal a salient super-HSA (HSA #2). Anchored by Lianzhou People’s Hospital and Liannan People’s Hospital, this area’s markedly greater service capacity exerts a strong pull, with a catchment extending beyond its administrative jurisdiction and drawing patients from outside the county. This phenomenon produces spatial disequilibrium in healthcare resources: villages proximate to the super-HSA but administratively external (e.g., Dalong and Neitian) exhibit high rates of cross-region care-seeking, functionally decoupling them from their administratively assigned health-service system and rendering them edge village. Consequently, the super-HSA shapes edge villages from both the supply and demand sides: on the supply side, it accentuates the relative insufficiency of primary-level services, reinforcing a core–periphery gradient; on the demand side, it shifts patients’ care-seeking calculus, making cross-region pursuit of higher-quality care common.

#### Mismatch between functional regions and administrative boundaries

Except for super-HSA(#2), the HSAs do not form independent service areas strictly along administrative boundaries. This suggests that the township health centers are insufficient to fully meet the needs of permanent residents, and there is a certain degree of mismatch between actual HSA boundaries and administrative boundaries.

Finally, the supply–demand situation of the seven HSAs, identified based on patient flow data, is presented in Table 2.

**Table 2:** Supply and demand situation of each HSA

HSA	Population (persons)	Service providers within liannan county	Service providers outside liannan county
#1	40,271	Zhaigang township health center, Zhaigang township health center Zhainan branch	None
#2	224,931	Liannan people’s hospital Sanjiang health center, Sanpai health center	Lianzhou people’s hospital, Lianzhou township health center
#3	6905	Damaishan township health center	None
#4	8526	Woshui township health center	None
#5	15,109	Daping township health center	Taibao township health center
#6	62,160	Xiangping township health center	Lianshan people’s hospital, Futang township health center, Jitian township health center
#7	53,196	None	Jiubei township health center, Libu township health center

#### Identification of edge villages

**Fig. 7:**
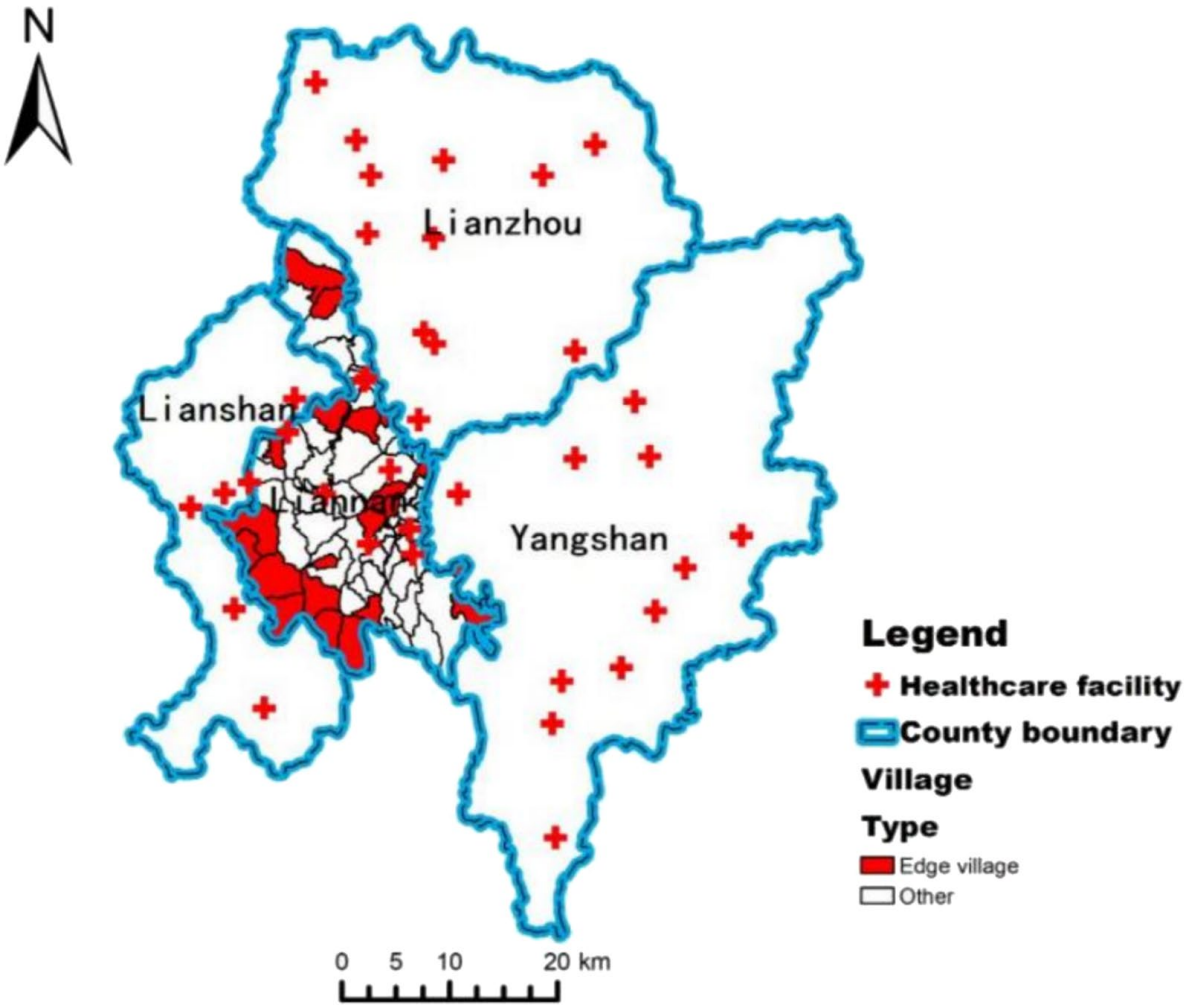
Spatial distribution of edge villages

Based on the analysis of the HSAs derived from the Louvain algorithm, this study defines villages that are assigned to other administrative regions and exhibit distinct cross-region healthcare-seeking behavior as edge villages. The identified edge villages are listed in Table 3, and they were visualized in ArcGIS and marked in Fig. 7.

**Table 3:** List of edge villages

Community name	Township	Main outflow destination
Shangdong village	Damaishan township	Zhaigang central health center
Wangjialing village	Damaishan township	Zhaigang central health center
Tanglicheng village	Damaishan township	Zhaigang central health center
Dazhang village	Daping township	Liannan People’s hospital
Niulushui village	Daping township	Liannan people’s hospital
Dalong village	Sanjiang township	Lianzhou peopl’s hospital
Neitian village	Sanjiang township	Lianzhou people’s hospital
Baijindong village	Sanjiang township	Zhaigang central health center
Shanxi village	Sanpai township	Liannan people’s hospital
Wugongtian village	Sanpai township	Zhaigang central health center
Niutouling village	Sanpai township	Liannan people’s hospital
Hengkeng village	Sanpai township	Zhaigang central health center
Dazhuwan village	Woshui township	Liannan people’s hospital
Panshi village	Xiangping township	Futang township central health center
Paidu village	Xiangping township	Futang township central health center
Longshui village	Xiangping township	Jitian township central health center
Tangqier village	Xiangping township	Jitian township central health center
Shanlian village	Zhaigang township	Lianzhou people’s hospital

### Determinants of the edge village formation

**Fig. 8:**
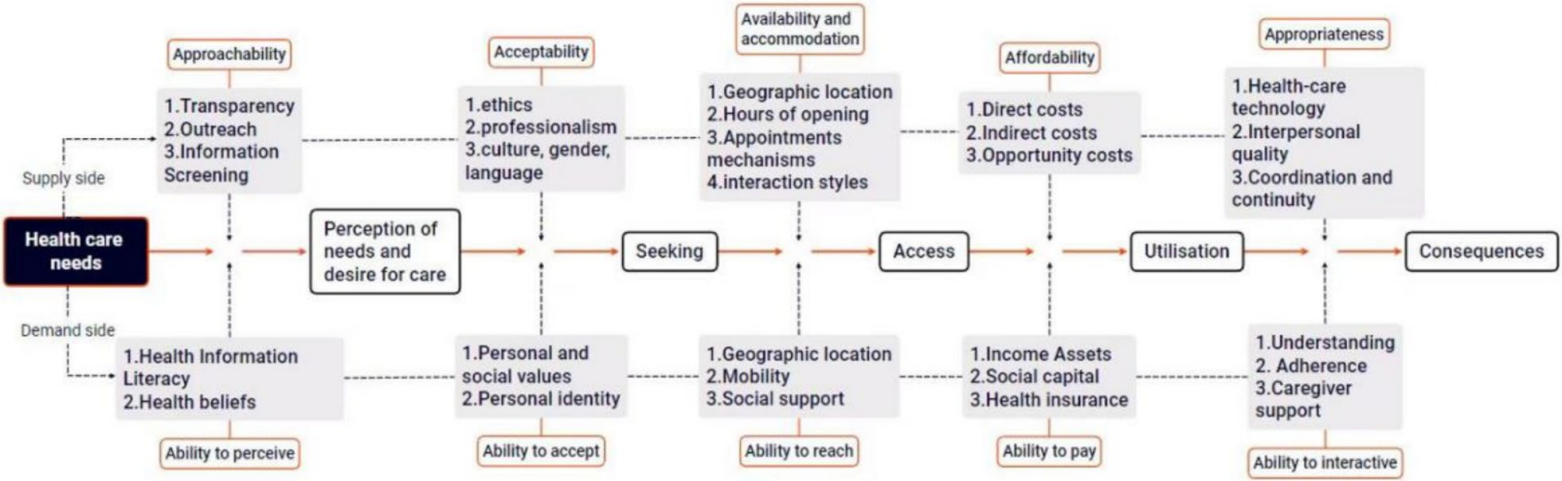
Factors influencing residents’ healthcare choices and cross-region healthcare behavior

To identify factors shaping the formation of edge villages, we conducted a structured literature search. We searched Web of Science and China National Knowledge Infrastructure (CNKI) using Boolean combinations of the following keywords: “patient mobility,” “interregional mobility,” “out-of-area healthcare,” “bypass care,” “cross-border healthcare,” “care selection,” and “accessibility.” We also performed backward- and forward-citation snowballing to identify additional studies. Figure 8 synthesizes the resulting set of candidate determinants.

Residents’ healthcare choices result from the interplay of individual, family, social, environmental, and healthcare system characteristics. This study focuses on the healthcare-seeking behaviors commonly observed at the village level. Therefore, in selecting indicators, certain individual characteristics (such as education level, occupation, and health status) were excluded or aggregated into community-level characteristics (such as payment ability, age, and gender). Similarly, healthcare provider characteristics consider only basic resources and structures, excluding local policies. Additionally, all diagnostic, treatment-related, and individual-based clinical indicators were excluded from the analysis. Based on these considerations, the potential factors influencing the formation of edge villages are categorized into three types: healthcare system (or institutional) supply-side characteristics, community demand-side characteristics, and the relational characteristics between supply and demand. The corresponding factor framework is summarized in Table 4

**Table 4:** Influencing factors for the formation of edge villages

Type	Factors	Main references
Demand-side characteristics	1. Total demand	[3, 33]
	2. Regional development status	[3, 32]
	3. Per capita economic status	[3, 32, 34, 50, 52]
	4. Per capita government health expenditure	[23, 39, 77]
	5. Population age structure	[3, 21, 31, 34, 52]
	Population ethnic composition	[25]
	Transportation conditions	[12, 33, 37, 63]
	Residential terrain	[12]
	Population distribution	[57, 70]
Supply-side characteristics	10.Regional development status	[3, 21, 46]
	Medical service costs	[9, 17, 37]
	(Perceived/objective) service capacity	[12, 17, 33, 37, 69]
	(Perceived/objective) healthcare quality	[14, 37, 63]
	Regional organizational structure	[3, 27, 28, 42, 67]
	Pharmaceutical and medical technology level	[3, 34, 37, 69]
Relational characteristics	Geographical distance	[1, 3][25, 31, 34, 50]
	Political similarity	[1, 3]
	Linguistic and cultural similarity	[3, 38],Zhang 2022; [2, 11, 19, 56]
	Trust in doctors	[17, 37, 60]

### Selection of key determinants

#### Preliminary screening and indicator selection

Based on the practical conditions of remote rural areas, in conjunction with a literature review and the current healthcare situation in Liannan County, this study applies the following principles to select representative indicators: (1) Feasibility and data availability: The indicator should be practically applicable in the context of remote and underdeveloped areas; (2) Quantifiability: The indicator should be measurable to facilitate subsequent computational analysis; (3) For indicators involving both supply and demand sides, a comprehensive approach is adopted. According to these criteria, 19 identified factors were consolidated into 12 representative indicators (see Table 5).

**Table 5:** List of representative indicators

Type	Core factors	Representative indicators
Demand-side characteristics	Total demand	Population size
	Regional development status	2. Village classification
	3. Population age structure	3. Population age structure
	4.Population ethnic composition	4. Population ethnic composition
	5. Transportation conditions	5. Distance to national or provincial roads
		6. Bus services frequency
	6. Residential terrain	7. Elevation
		8. Slope
	7. Population distribution	9. Cohesion index
Supply-side characteristics	8. (Perceived/objective) service capacity	10. Facility service capacity scale
	9. (Perceived/objective) healthcare quality	
	10.Pharmaceutical and medical technology level	
Relational characteristics	11. Geographical distance	11. Travel time
	12. Political similarity	12.Cross-county healthcare-seeking
	13. Linguistic and cultural similarity	

Through field surveys and a literature review, this study identifies two patterns of cross-region healthcare mobility behaviors [10, 47]: The first pattern, referred to as passive mobility, occurs when the local healthcare service supply is insufficient to meet demand. This is particularly common for common diseases and results mainly from an imbalanced local service supply structure. Additionally, long-distance cross-region healthcare for rare, complex, or severe conditions (such as organ transplants or pediatric surgeries) also falls under the category of inadequate supply. However, since investigating these situations requires a more specialized clinical background and involves broader service coverage and more complex research dimensions, they are not the primary focus of this study. The second pattern, referred to as active mobility, arises from relative deficiencies in service supply. This is primarily characterized by patients actively selection healthcare services based on comparative advantages, such as proximity, public transportation convenience, or higher levels of medical resources and technological expertise.

The 12 indicators in Table 5 are classified into two groups according to their primary attributes. The indicators for active mobility include travel time, facility service capacity, and cross-region healthcare. The indicators for passive mobility include population size, village classification, population age structure, population ethnic composition, distance to national or provincial roads, bus Services frequency, elevation, slope, and cohesion index. While indicators such as village classification and the cohesion index can influence—and be influenced by—both supply- and demand-side dynamics, our classification follows Andersen’s Behavioral Model, which treats community-level attributes as enabling factors shaping care-seeking from the demand side. This perspective emphasizes the primary pathway through which these variables operate in our analysis, while recognizing potential bidirectionality. Further research is warranted to disentangle these interactions empirically.

#### Active mobility indicators

Travel time

##### Travel time

Given the characteristics of rural and mountainous areas in Liannan County, this study utilizes the AutoNavi Map Web Service Path Planning API to calculate travel time from residential areas to healthcare facilities as a measure of spatial accessibility. A car was chosen as the reference mode of transportation because its travel time estimates can be scaled to other modes, providing strong applicability. The geographic location of villages was represented by population-weighted centroids to improve the spatial accuracy in rural areas. To minimize errors, travel time data were recorded every two hours starting at 6:00 AM on June 1, June 15, and July 1, 2023. After removing outliers, the mean value was calculated, yielding 4,544 data points covering 71 villages and 64 healthcare facilities.

##### Facility service capacity

This study assesses the comprehensive service capacity of healthcare institutions using factor analysis, incorporating indicators such as total wage expenditures, the number of practicing physicians, nurses, and health technicians. Data were obtained from the official website of the Linnan County Health Bureau and official reports published by healthcare institutions in 2021. Common indicators, such as the number of beds and occupancy rate, were excluded, as field research indicated that inpatient services in rural primary healthcare institutions are relatively scarce.

##### Cross-county healthcare-seeking

This indicator measures the barriers associated with cross-county healthcare-seeking. Seeking medical care across county borders may pose communication challenges due to languages and cultural differences. In this study, a binary county boundary variable is used, assigned a value of 1 if the healthcare-seeking choice crosses a county boundary and 0 otherwise.

#### Passive mobility indicators

##### Population size

This indicator reflects the intrinsic demand for healthcare services in a given area. Regions with larger populations benefit from economies of scale, which can reduce per capita healthcare costs and enhance service availability, thereby reducing patient outflow. Data were obtained from the respective township governments in Linnan County.

##### Village classification

This indicator is closely related to regional fiscal and healthcare expenditures, which influence the efficiency of the healthcare system and its service capacity. In this study, villages were classified into three levels (Excellent = 3, Good = 2, Poor = 1) based on village development plans, gross township economic output, and average farmer income levels.

##### Population age structure

This indicator is measured by the proportion of the population aged 60 and above. The elderly population is more sensitive to both economic and time-related costs, which affects their healthcare-seeking behavior. Data were obtained from the township-level population census.

##### Population ethnic composition

The proportion of Yao ethnic group reflects the healthcare preferences of minority populations, who tend to prefer healthcare providers from the same ethnic group due to cultural and language differences. Data were collected through telephone follow-ups and surveys.

##### Distance to provincial and national roads, and bus service frequency

Transportation conditions play a crucial role in shaping healthcare choices. Since station names do not always accurately reflect the actual coverage of bus routes, this study adjusted for this discrepancy: villages within a 5-min walking distance from a bus stop were included in the coverage area of the relevant bus route. Road network data were obtained from AutoNavi Map and the official website of the Liannan Yao Autonomous County Transportation Bureau (Liannan Yao Autonomous County Transportation [35]).

##### Elevation and slope

Patients in mountainous areas are more likely to utilize primary healthcare institutions. This study used the 1:10,000 DEM of Liannan County to calculate the elevation and slope of villages using ArcGIS, applying area-weighted averaging.

##### Cohesion index

This index reflects the degree of aggregation or dispersion in population distribution, considering the impact of residential building spatial patterns on healthcare choices. The index ranges from −1 to 1, where −1 indicates complete dispersion, 0 represents a random spatial pattern, and 1 indicates a highly clustered distribution. The formula is as follows:

6 $$\begin{array}{c}COHESION=\left(1-\frac{\sum_{j=1}^{m}{P}_{j}}{\sum_{j=1}^{m}{P}_{j}\sqrt{{a}_{j}}}\right){\left(1-\frac{1}{\sqrt{A}}\right)}^{-1}\end{array}$$

where: ${P}_{j}$ represents the perimeter of building patch $j$; ${a}_{j}$ represents the area of building patch $j$; m represents the number of building patches in each village; A represents the total building coverage area in each village.

Data were obtained from the planning land use dataset provided by the Liannan Yao Autonomous County Natural Resources Bureau.

#### Selection of key indicators

This study employs the Elastic Net regression model to screen the 12 representative indicators summarized earlier. Using the Elasticnet Logit command in Stata software, tenfold cross-validation was applied to select the optimal regularization parameters λ and α. The cross-validation results are shown in Table 6. The model achieves the smallest error when α = 0.5 and λ = 0.034573, indicating a balance between variable selection and model fitting. Meanwhile, the model selected 7 variables (as shown in Table 7), including 3 active mobility variables: facility service capacity, travel time, and cross-county healthcare-seeking; and 4 passive mobility variables: village classification, population ethnic composition, population size, and cohesion index.

**Table 6:** Selection of parameters α and λ

Alpha	ID	Description	Lambda	No.of nonzero coef	Out-of-sample dev. ratio	CV mean deviance
1						
	1	First lambda	222.2369	0	0.0106	1.400954
	111	Last lambda	0.013636	12	0.159	1.165811
0.9						
	112	First lambda	222.2369	0	0.0106	1.400954
	221	Last lambda	0.014966	12	0.1598	1.164819
0.8						
	222	First lambda	222.2369	0	0.0106	1.400954
	330	Last lambda	0.016425	9	0.1604	1.163906
0.7						
	331	First lambda	222.2369	0	0.0106	1.400954
	438	Last lambda	0.018026	8	0.161	1.163057
0.6						
	439	First lambda	222.2369	0	0.0106	1.400954
	544	Last lambda	0.021713	8	0.1619	1.161823
0.5						
	545	First lambda	222.2369	0	0.0106	1.400954
	644	Lambda before	0.037944	6	0.1641	1.158753
	* 645	Selected lambda	0.034573	7	0.1643	1.158593
	646	Lambda after	0.031502	8	0.1637	1.159334
	649	Last lambda	0.02383	8	0.1622	1.161374
0.4						
	650	First lambda	222.2369	0	0.0106	1.400954
	753	Last lambda	0.026153	8	0.1623	1.16136
0.3						
	754	First lambda	222.2369	0	0.0106	1.400954
	859	Last lambda	0.021713	9	0.1592	1.165657
0.2						
	860	First lambda	222.2369	0	0.0106	1.400954
	963	Last lambda	0.026153	9	0.1591	1.165795
0.1						
	964	First lambda	222.2369	0	0.0106	1.400954
	1066	Last lambda	0.028703	9	0.1568	1.168875
0						
	1067	First lambda	222.2369	12	0.0101	1.400329
	1246	Last lambda	2.22E-05	12	0.1185	1.221977

**Table 7:** Key indicators selected by the elastic net regression model

Indicator category	Key indicator	Variable name	Standard deviation	Mean
Active mobility	Travel time	D	993.3495	1404.782
	Facility service capacity	S	250.9081	386.831
	Cross-county healthcare-seeking	B	0.459477	0.302817
Passive mobility	Population size	P	2233.484	1897.07
	Village classification	T	0.906894	2.225352
	Population ethnic composition	N	0.413282	0.605973
	Cohesion index	C	0.032263	0.859426

## Discussion

To investigate cross-region healthcare choice behavior, this study employs a mixed logit model, which accommodates both alternative-specific variables and individual-specific variables. The alternative-specific variables (whose values vary across alternative choices) included Travel Time, Facility Service Capacity, and Cross-county Healthcare. The individual-specific variables (whose values remain constant across all choices for a given decision-maker) encompassed community-level characteristics such as the Cohesion Index and Village Classification. The model’s observable utility function of is as follows:

7 $$\begin{array}{c}{V}_{i{j}_{0}}=ASC+{\beta }_{1}{\text{Distance}}_{ij}+{\beta }_{2}{\text{Scale}}_{ij}+{\beta }_{3}{\text{Cohesion}}_{ij}+\\{\beta }_{4}{\text{Type}}_{ij}+{\beta }_{5}{\text{Population}}_{ij}\\ +{\beta }_{6}{\text{Nationality}}_{ij}\end{array}$$

To ensure model parsimony and efficacy, we conducted a systematic diagnosis of the initially included "Cross-county Healthcare" variable. The decision to exclude it from the final model was based on a triad of evidence: (1) Lack of Statistical Significance: The variable’s coefficient estimate had a high P-value of 0.572 (well above the 0.1 significance threshold), indicating a statistically insignificant effect; (2) Likelihood Ratio Test Favors Simpler Model: Comparing models with and without the variable, the likelihood ratio test yielded χ^2^(1) = 0.26 (P = 0.613), confirming that omitting it did not significantly degrade the model’s fit, thus justifying the simpler specification; (3) Multicollinearity Ruled Out: The variable’s Variance Inflation Factor (VIF) value was 1.15, significantly below the common threshold of 10, confirming that its insignificance was not due to multicollinearity with other explanatory variables.

This evidence collectively assures that omitting this variable avoids omitted variable bias while yielding a more parsimonious and efficient model. Parameters for the final model were estimated using maximum likelihood estimation in STATA. The results show that the model’s Wald chi2 statistic is significant at the 5% level (Prob > chi2 = 0.0135), indicating the model is overall valid. The calibration results are presented in Table 8.

**Table 8:** Parameter estimates of the mixed logit model

Variable type	Variable name	Coefficient	Standard error	P>z
Alternative-specific				
	Travel time	−0.0033925***	0.0010108	0.001
	Facility service capacity	0.0054233*	0.0032124	0.091
Individual-specific(For alternative #1: cross-region)				
	Cohesion index	33.47307*	18.42857	0.069
	Village classification	−1.420579**	0.7339432	0.05
	Population size	0.0002493*	0.0001784	0.075
	Population ethnic composition	4.166709*	2.454704	0.09
	Constant	−27.25859*	15.81025	0.085
Alternative #2: Non-cross-region		(Base alternative)		
Model fit statistics				
Wald chi²(6)		16.04		
Prob >chi²		0.0135		

*** denotes P < 0.01, ** denotes P < 0.05, * denotes P < 0.1

### Impact of active mobility indicators

In the mixed logit model, mode #1 represents the choice of non-cross-region healthcare services. The interpretation of each indicator is as follows: a positive coefficient indicates a higher likelihood of selecting non-cross-region healthcare, whereas a negative coefficient suggests a greater tendency toward cross-region healthcare.

#### Travel time

**Fig. 9:**
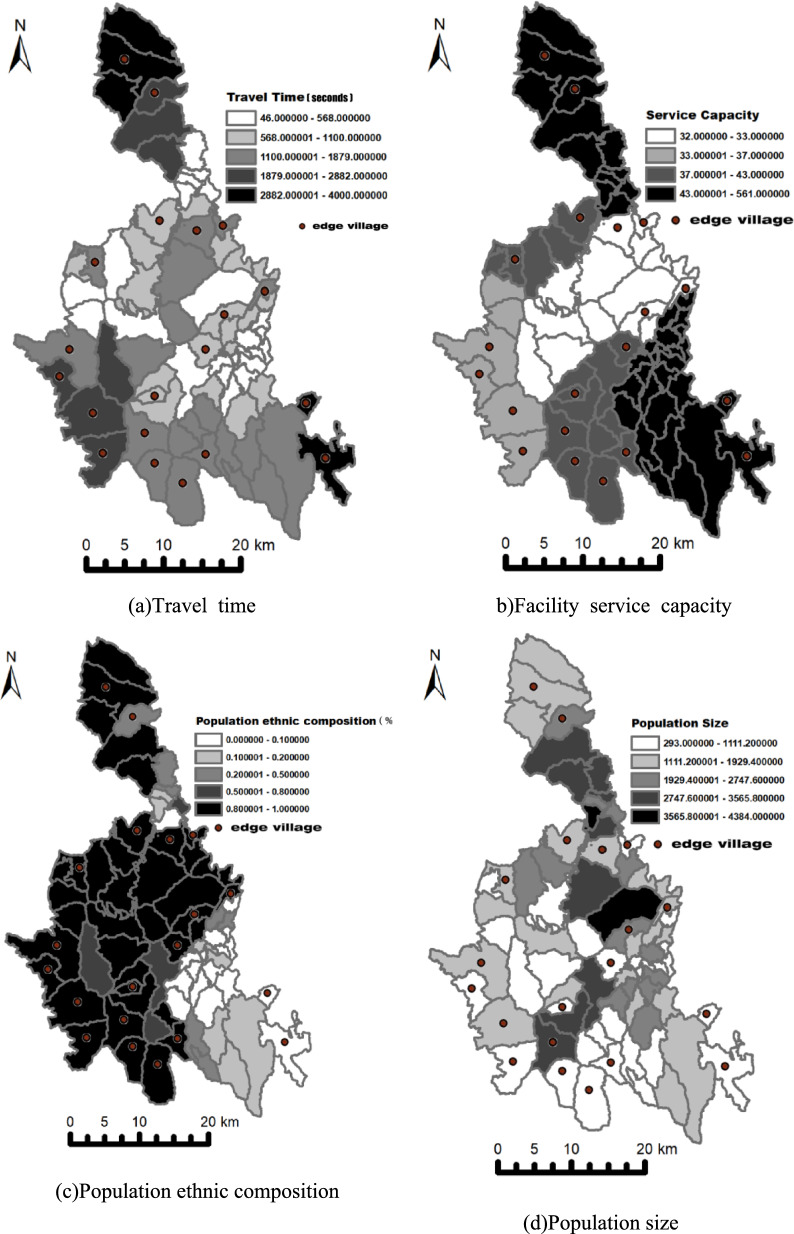

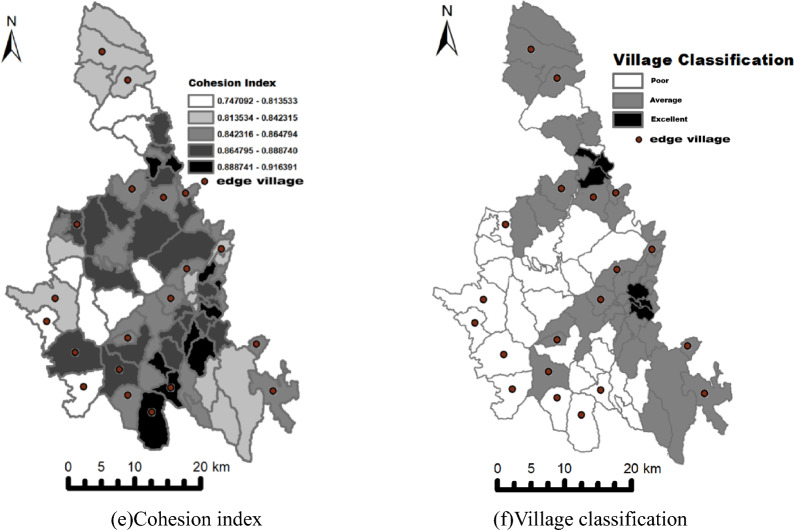
Spatial distribution of key indicators

The longer the travel time to local health-service facilities, the more likely a village is to become an edge village. As shown by the spatial pattern of travel time (Fig. 9a), villages with the longest travel times almost invariably emerge as edge villages, directly reflecting the critical role of geographic distance in healthcare accessibility. Specifically, each additional second of travel time reduces the probability of choosing the non–cross-region option by 0.34% (exp (− 0.033925)). This indicates that, in remote areas, greater distance markedly diminishes residents’ preference for local facilities.

#### Facility service capacity

Facility service capacity has a positive coefficient, indicating that stronger capacity (e.g., more health technicians, higher health expenditures) increases the likelihood that residents choose local providers. The spatial configuration of capacity (Fig. 9b) further corroborates this pattern: edge villages are more likely to occur in low-value zones and across sharp capacity discontinuities. Specifically, each one-unit increase in the attraction index (our proxy for facility service capacity) raises the probability of choosing the non-cross-region option by 0.54% (exp (0.033925)). This finding underscores the pivotal role of strengthening service capacity and quality in retaining patients and reducing passive outflows.

### Impact of passive mobility indicators

Among community-level characteristics, population size, population ethnic composition, and the cohesion index all positively influence residents’ likelihood of utilizing local healthcare facilities, whereas village classification has a negative effect. The mechanisms underlying these indicators are elaborated below.

#### Population ethnic composition

Villages with a higher Yao population share are more likely to use local providers. In the population ethnic composition map (Fig. 9c), areas with a larger Yao share exhibit fewer edge villages, a pattern consistent with cultural- and language-concordance effects. Within local communities, Yao residents may benefit from familiar linguistic support and culturally concordant settings, which can lower barriers to local care-seeking.

#### Population size

Population size has multifaceted effects, but in our setting larger communities are, on balance, more likely to use local facilities. The population size map (Fig. 9d) shows that larger villages are less likely to be classified as edge villages. Greater population size typically augments fiscal and human resources to support service systems and infrastructure; higher demand can generate economies of scale, lowering unit costs, improving efficiency, and expanding service scope. By contrast, limited demand can undermines financial sustainability, reducing local availability and diversity of care. In more populous settings, greater provider competition may emerge, potentially raising service quality. Taken together, these factors imply a negative association between population size and patient outflows.

#### Cohesion index

Although some studies posit that more concentrated communities have a broader care-seeking radius, our estimates indicate the opposite. The cohesion index map (Fig. 9e) shows that low-cohesion areas are more prone to edge villages. A plausible explanation is that, in more concentrated settings, healthcare demand is more spatially focused, service systems are more complete, and facilities are on average closer, jointly reducing patient outflows.

#### Village classification

Development level is negatively associated with choosing local care—that is, higher development is associated with greater cross-region choice. The spatial pattern (Fig. 9f) likewise suggests that edge villages cluster in higher-development areas and along developmental-transition belts. A plausible explanation is that stronger local development and higher household incomes increase mobility and ability to pay, broadening the feasible geographic choice set.

### Policy implications: evidence-informed, targeted governance pathways

To alleviate cross-region care-seeking among residents of edge villages and reduce inequities in the spatial distribution of healthcare resources, we propose the following measures.

#### Recognize the super-HSA attraction and establish a tiered coordination framework

The existence of a super-HSA reflects the agglomeration of high-quality medical resources; policy should channel this influence through an institutionalized, tiered coordination framework. Specifically, strengthen formal care-coordination networks between core hospitals in the super-HSA and primary-level facilities in edge villages; standardize two-way referral criteria and interoperable care pathways. In parallel, encourage core hospitals to extend high-quality services to edge villages via tele-consultation, outreach clinics, and technical assistance, aligning with observed patient flows to enhance the resilience of the regional care network.

#### Strengthen local service capacity to mitigate the super-HSA siphoning effect

Given the positive association between facility service capacity and patient retention, and the siphoning pull of the super-HSA, strengthening local capacity in edge villages is essential. Implement targeted assistance programs, mobile clinics and outreach services, and workforce-incentive policies to reinforce the core competencies of village health stations, particularly for common conditions and chronic-disease management. Ensure reliable provision of essential medicines and basic diagnostic equipment, thereby increasing residents’ confidence in local care and reducing passive patient outflows driven by capacity shortfalls.

#### Systematically reduce the composite costs of cross-region care-seeking

Travel time is the strongest negative determinant of local-care choice. Accordingly, time costs should be reduced systemically. On one front, optimize rural transit networks by adding routes that connect directly to functional care hubs (HSAs) rather than merely to administrative seats; In parallel, scale up telemedicine in edge villages and establish convenient access points, converting physical distance into instant digital connectivity and thereby compressing care-seeking time.

#### Strengthen community support and culturally concordant services

Given the significant effects of community attributes (e.g., ethnic composition and population size), policy should prioritize building community resilience. In minority-concentrated villages, provide linguistically and culturally concordant care (e.g., deploy bilingual clinicians). In parallel, expand family-physician contracting and community health-volunteer networks to enhance residents’ attachment to and easy access to local services, thereby reducing demand-side incentives for cross-region care arising from cultural barriers or information asymmetries.

#### Implement dynamic governance of functional HSAs

We identify a mismatch between administrative borders and de facto functional care-seeking regions. We therefore recommend establishing a mechanism to dynamically adjust functional service-area (HSA) boundaries. Routinely use patient-flow data to identify and delineate actual service regions—such as the super-HSA—and use these delineations as inputs to regional health planning, fiscal allocation, and performance assessment. Without altering administrative authority, encourage cross-jurisdictional collaboration platforms to optimize resource allocation and harmonize health-insurance settlement processes, thereby aligning management boundaries more closely with observed care-seeking patterns.

## Conclusion

Using Liannan County as an empirical case, this study introduces and validates the concept of edge villages as a micro-analytic unit and a framework for analyzing the spatial misallocation of rural healthcare resources. The framework indicates that, when administrative borders are misaligned with de facto functional HSAs, cross-region care-seeking is not incidental but a systemic outcome of hierarchical resource distributions, individual choice, and institutional boundaries. This perspective enables a village-scale understanding of how inequities in rural healthcare allocation emerge.

On the theoretical side, the study ontributes to the literature in three ways. First, it moves beyond reliance on administrative units by introducing an analytical framework grounded in functional care linkages, shifting inquiry from macro regions to the village level and enabling precise identification of service-disadvantaged populations along administrative edges. Second, it develops an institution-behavior-space integrative framework that coherently combines boundary/edge-effect theory, Andersen’s behavioral model, and accessibility theory, offering a unified tool to explain the formation of edge villages. Third, it operationalizes the concept through an identification and estimation strategy, enabling quantification and laying a methodological foundation for subsequent comparative and longitudinal research.

Practically, the study provides an evidence base for pinpointing service gaps and optimizing county-level healthcare configurations. Building on the empirical findings, it proposes a systematic, multi-dimensional governance pathway for edge villages—service coordination, capacity building, cost reduction, community resilience, and dynamic governance. The results inform more rational resource allocation, the reconfiguration of service catchments and tiers, and cross-region health-insurance coordination; they also offer geographers and urban-regional planners a new analytical framework for incorporating edge-village care needs into planning and regional-development strategies, thereby promoting coordinated and equitable development.

Several limitations warrant mention. The evidence is drawn from a single-year, single-county dataset, constraining sample size and temporal coverage and potentially limiting generalizability. The data originate from a government-led early-cancer screening project, which—while mitigating selection bias to some extent—may over-represent health-conscious participants; although this feature aligns with a study of care-seeking behavior, caution is warranted when extrapolating to the entire population. Seasonal variability in mountainous transport conditions may also affect the stability of results. n addition, our classification of active versus passive mobility indicators—though theory-driven—inevitably overlaps: certain variables (e.g., village classification, cohesion index) encode both supply- and demand-side signals. We classify by the primary causal pathway to serve the paper’s analytic focus, while acknowledging this crossover and leaving scope for future work to disentangle these mechanisms with finer-grained data and causal-identification strategies.

These caveats point to directions for further research: (i) extending to multiple periods and regions to assess the stability and applicability of the edge-village concept; (ii) incorporating richer care-seeking and travel data, where feasible, to validate and refine our findings; and (iii) piloting service-area adjustments and cross-jurisdictional coordination at the county level to evaluate policy effectiveness and translate research into practice.

Notwithstanding these limitations, the edge village framework developed here offers a useful analytical lens and methodological support for understanding spatial mismatches in rural healthcare. We expect this work to deepen understanding of healthcare equity and to provide practical references for health-system reform and regionally coordinated development in comparable settings.

## Data Availability

The authors do not have permission to share data.
